# Ehretiquinone from *Onosma bracteatum* Wall Exhibits Antiaging Effect on Yeasts and Mammals through Antioxidative Stress and Autophagy Induction

**DOI:** 10.1155/2021/5469849

**Published:** 2021-01-13

**Authors:** Yanjun Pan, Yanan Liu, Rui Fujii, Umer Farooq, Lihong Cheng, Akira Matsuura, Jianhua Qi, Lan Xiang

**Affiliations:** ^1^College of Pharmaceutical Sciences, Zhejiang University, Yu Hang Tang Road 866, Hangzhou 310058, China; ^2^Department of Biology, Graduate School of Science, Chiba University, Chiba 263-8522, Japan; ^3^Faculty of Pharmacy, University of Central Punjab, Lahore 54660, Pakistan

## Abstract

The antiaging benzoquinone-type molecule ehretiquinone was isolated in a previous study as a leading compound from the herbal medicine *Onosma bracteatum* wall. This paper reports the antiaging effect and mechanism of ehretiquinone by using yeasts, mammal cells, and mice. Ehretiquinone extends not only the replicative lifespan but also the chronological lifespan of yeast and the yeast-like chronological lifespan of mammal cells. Moreover, ehretiquinone increases glutathione peroxidase, catalase, and superoxide dismutase activity and reduces reactive oxygen species and malondialdehyde (MDA) levels, contributing to the lifespan extension of the yeasts. Furthermore, ehretiquinone does not extend the replicative lifespan of *Δsod1*, *Δsod2*, *Δuth1*, *Δskn7*, *Δgpx*, *Δcat*, *Δatg2*, and *Δatg32* mutants of yeast. Crucially, ehretiquinone induces autophagy in yeasts and mice, thereby providing significant evidence on the antiaging effects of the molecule in the mammalian level. Concomitantly, the silent information regulator 2 gene, which is known for its contributions in prolonging replicative lifespan, was confirmed to be involved in the chronological lifespan of yeasts and participates in the antiaging activity of ehretiquinone. These findings suggest that ehretiquinone shows an antiaging effect through antioxidative stress, autophagy, and histone deacetylase Sir2 regulation. Therefore, ehretiquinone is a promising molecule that could be developed as an antiaging drug or healthcare product.

## 1. Introduction

Human beings demand a healthy life but are vulnerable to age-related diseases, including Alzheimer's disease, diabetes, cancer, and cardiovascular diseases [1]. With the continuous aging of the global population, the medical expenditures for the elderly increase the burden for their families and society. Therefore, effective therapy for controlling and preventing age-related diseases must be developed. As a vital risk factor of age-related diseases, aging refers to the degradation of an organism as characterized by the gradual accumulation of damaged substances [2]. These substances are triggered by oxidative stress and eliminated by autophagy; some genes, specifically the silent information regulator 2 (*SIR2*), respond to oxidative stress and improve aging [3, 4]. Oxidative stress is caused by the disequilibrium between the production of reactive oxygen species (ROS) and antioxidant systems. Although a low level of ROS is necessary to conduct a normal physiological function, the superfluous level of ROS can impair cellular lipids, proteins, or DNA and consequently produce harmful materials [5]. ROS can also be scavenged by catalase (CAT) and glutathione peroxidase (GPx), which are antioxidant enzymes that contribute to either extending or shortening the lifespan of an organism [5, 6]. Moreover, if the damaged substance is eliminated in time, then its negative effects on physiological functions can be minimized. As a highly conserved metabolic process, autophagy in organisms significantly contributes to the removal of damaged molecules. The damaged organelles or proteins are delivered to the lysosome after inducing autophagy and then degraded and recycled to maintain cellular homeostasis, which contributes to the longevity of organisms [7]. Previous studies showed that inducing autophagy alleviated the toxicity caused by oxidative stress while the defects of this process promoted oxidative stress, thereby highlighting the relationship and potential effect of autophagy on the aging mechanism of organisms [8]. The Sir2, which is an evolutionarily highly conserved NAD^+^-dependent deacetylase, can prevent senescence by protecting ribosomal DNA stability [9]. The deficiency and overexpression of the *SIR2* gene can shorten and extend the replicative lifespan, respectively [10]. However, whether *SIR2* also shows the same effect on the chronological lifespan remains debated [11, 12].

Yeast models have been extensively used in aging studies due to their relatively simple and short life cycle; a small genome comprising 6000 genes has been completely sequenced and mapped [13], and the ortholog of approximately 30% of yeast genes relates to human diseases [14]. Among the available yeast models, the K6001 yeast strain is uniquely characterized by the growth of only mother cells in the glucose medium [15], thereby facilitating the replicative lifespan evaluation of yeast while screening active compounds. Therefore, the K6001 yeast mutant has been utilized as an antiaging screening bioassay system, and many antiaging compounds, including cucurbitacin B, parishin, cholesterol, nolinospiroside F, and phloridzin, have been isolated from natural products in previous studies [16–20].

*Onosma bracteatum* is a medicinal herb used in Asian countries due to its antileprotic, antibacterial, and anti-inflammatory functions [21] and its role in enhancing memory and immunity [22]. As an important part of the Unani system, *O. bracteatum* has been used to treat Alzheimer's disease, arrhythmias, and hypertension [22]. The K6001 bioassay screening system also highlights the antiaging activity of this herb. A series of antiaging molecules are isolated on the basis of this system, and their chemical structure and antiaging activity are described [23]. Moreover, the structure–activity relationship study suggests that ehretiquinone (EHR) shows the best performance in extending the replicative lifespan of K6001. Studying the antiaging mechanism of EHR is imperative because it is a benzoquinone-type molecule with a significant antiaging activity. The mechanism behind the antiaging effects of EHR is reported in this paper.

## 2. Materials and Methods

### 2.1. General

A preparative high-performance liquid chromatography (HPLC) system was equipped with two ELITE P-230 pumps and a UV detector. Optical rotations were performed by using a JASCO P-1030 digital polarimeter, and nuclear magnetic resonance (NMR) spectra were detected by a Bruker AV III-500 spectrometer (Bruker, Billerica, USA). The Agilent Technologies 6224A Accurate Mass TOF LC/MS system (Santa Clara, CA, USA) was used for the high-resolution ESI-TOF-MS analyses. Column chromatography was conducted by using silica gel (with 200 to 300 meshes, Yantai Chemical Industry Research Institute, Yantai, China) or reversed-phase C18 (Octadecylsilyl, ODS) silica gel (Nacalai Tesque, Kyoto, Japan).

### 2.2. Plant Material and Reagents

The plant material was purchased in Mansehra, Khyber Pakhtunkhwa, Pakistan, and identified by the associate professor Dr. Zafar Ullah Zafar from the Institute of Pure and Applied Biology of the Bahauddin Zakariya University in Multan, Pakistan. The voucher specimen (20170220) of the plant was preserved at the School of Pharmaceutics of Zhejiang University. The following reagents were purchased from the indicated suppliers: chemical reagents of HPLC (TEDIA, Rhode Island, USA) and analytical grades (Sinopharm Chemical Reagent Co. Ltd., Shanghai, China), resveratrol (RES) (J&K Scientific Ltd., Beijing, China), rapamycin (Solarbio, Beijing, China), DAPI dihydrochloride (DAPI) (Macklin, Shanghai, China), leupeptin (Macklin, Shanghai, China), and dimethyl sulfoxide (DMSO) (Sigma, Saint Louis, USA).

### 2.3. Isolation and Structural Elucidation of EHR

Dried plant material (1.5 kg) was crushed to powder and soaked in 100% methanol (CH_3_OH) for three days with constant shaking at room temperature. The obtained crude extract (120 g) was partitioned between ethyl acetate (EtOAc) and water. The active EtOAc layer sample (30 g) was concentrated before its separation by a silica gel open column eluted with *n*-hexane/CH_2_Cl_2_ (100 : 0, 80 : 20, 50 : 50, and 0 : 100) and CH_2_Cl_2_/CH_3_OH (98 : 2, 95 : 5, 90 : 10, 80 : 20, and 0 : 100). A total of 11 fractions were obtained. Fraction 3 (5.2 g), which was eluted with CH_2_Cl_2_/CH_3_OH (95 : 5), was further separated by a silica open column with *n*-hexane/CH_2_Cl_2_ (100 : 0, 50 : 50, 30 : 70, 20 : 80, 10 : 90, and 0 : 100) and CH_2_Cl_2_/CH_3_OH (95 : 5 and 0 : 100). Seven fractions were then obtained, and fraction 4 (400 mg), which was eluted with *n*-hexane/CH_2_Cl_2_ (0 : 100), was purified by the ODS open column with CH_3_OH/H_2_O (40 : 60, 50 : 50, 55 : 45, 57 : 43, 60 : 40, and 100 : 0). Among the five fractions obtained, fraction 3 (37.2 mg) was subjected to HPLC purification (C30-UG-5 (*Φ* 10 × 250 mm, Nomura Chemical), 68% methanol, flow rate: 3 mL/min, and detector: 210 nm) to yield EHR (18.7 mg, *t*_*R*_ = 22.1 min). The chemical structure of EHR was identified by comparing the MS, ^1^H NMR, and ^13^C NMR spectra and specific rotation data with the literature [24]: ^1^H NMR (500 MHz, CDCl_3_): *δ*_*H*_ = 6.84 (1H, d, *J* = 10.5 Hz, H-5′), 6.62 (1H, d, *J* = 8.7 Hz, H-6), 6.58 (1H, d, *J* = 2.95 Hz, H-3), 6.53 (1H, m, H-5), 6.52 (1H, d, *J* = 10.6 Hz, H-6′), 6.25 (1H, d, *J* = 16.1 Hz, H-8′), 5.65 (1H, d, *J* = 6.3 Hz, H-8), 5.49 (1H, d, *J* = 16.1 Hz, H-7′), 5.04 (1H, s, H-10′b), 4.97 (1H, s, H-10′a), 3.83 (1H, d, *J* = 6.3 Hz, H-7), 2.78 (1H, d, *J* = 19.3 Hz, H-10b), 2.52 (1H, d, *J* = 19.2 Hz, H-10a), 1.73 (3H, s, H-11′), 1.69 (3H, s, H-11); ^13^C NMR (125 MHz, CDCl_3_): *δ*_*C*_ = 195.3 (C − 1′), 193.2 (C-4′), 149.9 (C-4), 145.0 (C-1), 140.8 (C-9′), 139.1 (C-5′), 138.7 (C-6′), 137.4 (C-8′), 131.8 (C-9), 127.7 (C-2), 124.1 (C-7′), 122.6 (C-8), 119.3 (C-10′), 117.6 (C-6), 114.9 (C-5), 114.3 (C-3), 80.7 (C-3′), 55.3 (C-2′), 39.4 (C-7), 36.2 (C-10), 22.7 (C-11), 18.2 (C-11′). [*α*]_D_^16^ +1.01(c 0.12, MeOH); high-resolution ESI-TOF-MS *m/z* 371.1250, which is calculated for C_22_H_20_O_4_Na (M + Na)^+371.1254^.

### 2.4. Yeast Strains, Cell Lines, and Culture Medium

The K6001 derived from W303 was used along with *Δsod1*, *Δsod2*, *Δskn7*, *Δuth1*, *Δgpx*, *Δcat*, *Δatg2*, and *Δagt32* yeast strains with a K6001 background in the replicative lifespan assay. By contrast, the YOM36 derived from BY4742 was used along with the *Δsir2* and *Δuth1* yeasts of a YOM36 background in the chronological lifespan assay. Wild-type BY4741 and YOM38 containing plasmid pRS316-*GFP-ATG8* were used to investigate antioxidative stress and detect autophagy levels. All yeast strains were stored in a freezer at −30°C and validated by Professors Breitenbach (Salzburg University, Austria) and Matsuura (Chiba University, Japan). The genotypes of yeast strains and mutants in this study were described in the previous study [16].

The yeast culture media used in this work included galactose liquid medium (3% galactose, 2% peptone, and 1% yeast extract), glucose liquid medium (YPD) (2% glucose, 2% peptone, and 1% yeast extract), glucose medium agar plate (2% glucose, 2% peptone, 2% agar, and 1% yeast extract), and SD (2% glucose, 0.17% yeast nitrogen base without amino acids, and 0.5% ammonium sulfate (BD Ditco)). The liquid culture was performed by using a shaker at 28°C, and a solid culture was obtained in an incubator at 28°C.

The PC12 cell line, which was derived from rat pheochromocytoma cells, was purchased from the Type Culture Collection of the Chinese Academy of Sciences (Shanghai, People's Republic of China). PC12 cells were cultured in Dulbecco's Modified Eagle's Medium (DMEM) with high glucose (Cellmax, Beijing, China) supplemented with 10% horse serum (Cellmax, Beijing, China) and 7.5% fetal bovine serum (Solarbio, Beijing, China) in a 5% CO_2_ incubator at 37°C.

### 2.5. Replicative and Chronological Lifespan Assays

In the replicative lifespan assay, a K6001 yeast strain, which was stored in a freezer at −30°C, was taken out, washed thrice with phosphate buffer solution (PBS), inoculated in a liquid galactose medium, and incubated in a shaker for 24 h. The K6001 cells were collected by centrifugation at 1800×g for 10 min and washed thrice with PBS. Afterward, approximately 4000 cells calculated by a hemacytometer were evenly spread on the surface of the glucose medium agar plate containing RES at 10 *μ*M or EHR at doses of 0, 0.1, 1, 3, 10, and 30 *μ*M. The microcolony formation on the agar plates after cultivation at 28°C for 48 h was observed with a microscope. Forty microcolonies from each group were randomly selected, and the number of daughter cells produced by a mother cell in microcolonies was counted. The performance of the replicative lifespan assays of K6001 mutants (*Δsod1*, *Δsod2*, *Δuth1*, *Δskn7*, *Δgpx*, *Δcat*, *Δatg2*, and *Δatg32*) was similar to that of the K6001 strain. RES, as a well-known antiaging substance, was used as a positive control. Ethanol was used in the yeast experiment to dissolve compounds and as the vehicle for the control group. EHR did not affect the cell growth at doses of 1 to 30 *μ*M.

The chronological lifespan assay was conducted by following the described methodology [25]. The YOM36 or *Δsir2*, *Δuth1* of YOM36, was incubated in YPD by shaking for 24 h, transferred to a 20 mL SD medium, and incubated for another 24 h. On day 0, the yeast cell suspension was inoculated in a new 100 mL SD medium with an initial OD_600_ value of 0.01 and treated with EHR at concentrations of 0, 3, and 10 *μ*M with shaking at 28°C. On the third day, nearly 200 yeast cells were spread on the glucose medium agar plate with the stabilization of yeast growth, and the colony-forming units (CFUs) on the plates were counted after a two-day incubation. This process was undertaken every two days, and the EHR in different concentrations was added to the corresponding medium on day 7. The CFUs at day 3 were fixed as a 100% survival.

### 2.6. Yeast-Like Chronological Lifespan Assay

The yeast-like chronological lifespan assay was performed following the methods described in reference [26]. First, 80,000 PC12 cells were seeded in each well of a 96-well plate and cultured for 24 h. Then, the medium was replaced with 1 mL serum-free DMEM containing a test sample at different concentrations or DMSO of 0.5%. The cells were continually incubated for six days, and the medium was replaced every two days by a new serum-free DMEM containing doses of samples or DMSO (0.5%). Rapamycin inhibits the mTOR signaling pathway and has beneficial effects on the health span and lifespan of all cellular and organism systems [27]. Therefore, rapamycin was used as a positive control in the experiment. The cells treated in the 96-well plate were subsequently trypsinized, and a 5% aliquot was plated on fresh medium-filled six-well plates. Finally, the colony formation on the plate after 15 days was stained with crystal violet and photographed.

### 2.7. Antioxidative Stress Assay

The BY4741 yeast strain stored in the freezer at −30°C was taken out, washed thrice with PBS, inoculated in YPD, and incubated for 24 h. The BY4741 cells with an initial optical density at 600 nm (OD_600_) of 0.1 were inoculated in a 20 mL YPD containing the positive control, RES at 10 *μ*M, or EHR at 0, 3, and 10 *μ*M. After a 12 h cultivation, the yeast suspension of each group with an OD_600_ of 1.5 was dropped on the glucose medium agar plate containing 9.0 mM of H_2_O_2_ and continually incubated at 28°C for three days. The qualitative growth of yeast was also photographed.

A total of 200 cells from the treated or untreated groups were evenly spread on the glucose medium agar plate containing H_2_O_2_ at 0 or 5 mM and cultured for 48 h to obtain quantitative results. The CFUs were then counted to calculate the survival rate as follows: the number of cells survived in the 5 mM H_2_O_2_ divided by that survived in the H_2_O_2_ untreated group.

### 2.8. ROS and MDA Level Assays

For the ROS assay, BY4741 was cultured in 20 mL YPD for 23 or 47 h. Approximately, 5 × 10^7^ cells from each group were harvested and washed with PBS thrice and suspended in 1 mL of PBS. Afterward, 2′,7′-dichlorodihydrofluorescein diacetate (DCFH-DA) was added and mixed under dark conditions to reach a final concentration of 10 *μ*M. The suspended mixture was incubated by shaking for 1 h at 28°C, and yeast cells were collected and washed with PBS thrice. By using a SpectraMax M3 multimode microplate reader (Molecular Devices Corporation, California, USA), the DCF (2′,7′-dichlorofluorescein) fluorescence intensity of 1 × 10^7^ cells was recorded with excitation and emission wavelengths of 488 and 525 nm, respectively.

In the MDA assay, after culturing BY4741 in a 20 mL YPD for 24 or 48 h, all yeasts were harvested and washed with PBS thrice for 10 min at 1800×g and suspended in 500 *μ*L PBS. The yeast cells were ultrasonicated for 5 min, followed by five freezing cycles in liquid nitrogen for 5 min, soaking in a 37°C water bath for 2 min and another 5 min of ultrasonication. The cell lysates were centrifuged at 4°C for 10 min at 12,000×g to obtain the supernatant, which was then used to evaluate the MDA level by using the MDA assay kit (Nanjing Jiancheng Bioengineering Institute, Nanjing, China).

### 2.9. CAT, Total GPx, and SOD Activity Assays

Fig3 a, b, c, d, e and f.BY4741 yeast cells were cultured as described in the MDA assay for 24 h. These cells were then washed with PBS thrice, suspended in 250 *μ*L of PBS, and ultrasonicated on ice for 5 min. Afterward, these cells were centrifuged at 4°C for 10 min at 12,000×g to obtain a supernatant. Consequently, this supernatant measured the CAT, GPx, and superoxide dismutase (SOD) activities by, respectively, using CAT, GPx (Beyotime Biotechnology Limited Company, Shanghai, China), and SOD (Nanjing Jiancheng Bioengineering Institute, Nanjing, China) assay kits following the instructions of the manufacturer. For the CAT enzyme activity assay, different concentrations of hydrogen peroxide solution were first taken. Then, a color working solution was added to the CAT enzyme activity assay kit and reacted at 25°C for 15 min. The absorption value of A520 was measured, and the standard curve of hydrogen peroxide concentration was determined. Afterward, the catalase buffer and 250 mM hydrogen peroxide were added to each sample (5 *μ*g protein). The enzyme reaction termination solution was added after reaction at 25°C for 1–5 min to terminate the reaction. The color working solution was also added and reacted at 25°C for 15 min, and the absorption value of A520 was measured. For the GPx enzyme activity assay, 2 *μ*g of protein of each sample was taken, and the total glutathione enzyme assay kit was used to determine the glutathione enzyme activity. The general protocol is as follows: the GPx detection solution, samples, GPx detection working solution, and peroxide reagent were added in a 96-well plate in proper order. The absorbance value of A340 at every 4 min was measured seven times after mixing. For the SOD enzyme activity assay, 25 *μ*g of each sample protein was first mixed with reagent VII and reacted for 1 min to remove the Mn-SOD enzyme activity in the samples. The supernatant was then obtained as samples after centrifugation. The reagent I, blank control, samples, and the samples treated by reagent VII were added to the 96-well plate. Afterward, reagents II, III, and IV were added, mixed well, and incubated at 37°C for 40 min. Finally, the A550 absorbance value of samples was measured after adding color reagents and standing at room temperature for 10 min.

### 2.10. Fluorescence Image of Yeast Autophagy

The YOM38 yeast strain containing the pRS316-*GFP-ATG8* plasmid was primarily cultured in 20 mL YPD medium for 24 h. The yeast cells were then harvested and washed with PBS before inoculation in an synthetic deﬁned (SD) medium with an initial OD_600_ of 0.1 and treated with a positive control RES at 300 *μ*M and EHR at 0, 3, and 10 *μ*M. After cultivating for 22 h, the yeast cells were collected, washed, and suspended in 245 *μ*L PBS and stained with 5 *μ*L DAPI (1 mg/mL) in the dark for 10 min. These cells were then washed with PBS thrice and suspended in 10 *μ*L PBS to observe the differential interference contrast and the green and blue fluorescence images under two-photon confocal fluorescence microscopy (Olympus FV1000BX-51, Tokyo, Japan). The images were acquired and analyzed by using computer software (Olympus Fluoview Ver.4.1 Viewer). The light was avoided during the experiment.

### 2.11. Mice Autophagy Measurement

C57BL/6 mice were purchased from the Zhejiang Academy of Medical Sciences in Hangzhou, China. The autophagy assay was conducted by following the procedures of reference [26]. Six-week-old male C57BL/6 wild-type mice were injected intraperitoneally with 3 *μ*M EHR. The EHR was dissolved in 50 *μ*L DMSO before dilution with 150 *μ*L saline. The mice were treated with leupeptin in 200 *μ*L saline or vehicle saline after 4 h. The mice were then sacrificed after 2 h, and their liver and heart were collected. This animal experiment was performed in accordance with international ethical standards and was guided and approved by the Committee of Experimental Animal Care of Zhejiang University (permit number ZJU20190143).

Approximately, 100 mg of the liver tissue or 1 whole heart was homogenized in a lysis buffer containing a RIPA lysis buffer, 1% protease inhibitor and 1% cocktail 2 (CoWin Biotech, Beijing, China), and 1% cocktail 3 (Sigma, Saint Louis, USA). The homogenization was followed by a 20 min settlement, and the samples were centrifuged at 12,000×g for 10 min at 4°C. The supernatants were collected, and the protein concentration was measured by using the BCA protein assay kit (CoWin Biotech, Beijing, China). The proteins of the heart and liver tissues were used for the western blot analysis.

### 2.12. Real-Time Polymerase Chain Reaction (RT-PCR) Analysis

First, the wild-type BY4741 was incubated with EHR at a concentration of 0, 3, and 10 *μ*M in a glucose medium for 12 h at 28°C with shaking at 180 rpm. Total RNA was extracted using the hot phenol method. The reverse transcription method was employed to synthesize cDNA using the HiFi-MMLV cDNA Kit (Cowin Biotech, Beijing, P. R. China) and 5 ug of RNA. Quantitative RT-PCR was performed by using CFX96 Touch (Bio-Rad, Hercules, USA) and SYBR Premix EX Taq (Takara, Otsu, Japan) based on the previous study [16]. The thermal cycling parameters were as follows: 40 cycles, 94°C for 15 s, 60°C for 25 s, and 72°C for 20 s. The sequences of the primers for RT-PCR were as follows: for *SIR2*, sense 5′-CGT TCC CCA AGT CCT GAT TA-3′ and anti-sense 5′-CCA CAT TTT TGG GCT ACC AT-3′; for *TUB1*, sense 5′-CCA AGG GCT ATT TAC GTG GA-3′ and anti-sense 5′-GGT GTA ATG GCC TCT TGC AT-3′. The relative gene expression data were analyzed by the 2^-*ΔΔ*Ct^ method. The levels of *SIR2* mRNA were normalized to those of *TUB1.*

### 2.13. Western Blot Analysis

YOM38 yeast cells containing the pRS316-*GFP-ATG8* plasmid were treated with 300 *μ*M RES and EHR at 0, 1, 3, and 10 *μ*M for 22 h or with EHR at 3 *μ*M for 0, 8, 15, and 22 h to examine the changes in free green fluorescent protein (GFP) in yeast autophagy. All treated and untreated cells were harvested by centrifugation at 12,000×g for 10 min, washed thrice with PBS, and suspended in 150 or 250 *μ*L PBS. The yeast cells were collected, sonicated on ice for 5 min, and centrifuged to obtain the supernatant. The BCA assay kit was used to measure the concentration of proteins in the supernatant. Briefly, the 200 *μ*L BCA working solution and 25 *μ*L of each standard or sample were added into each well of 96-well plate. Each sample was repeated twice and mixed well. Subsequently, the 96-well plate was incubated at 37°C for 25 min, and the absorbance of the BSA standard and protein samples at 562 nm of wavelength was measured with the BioTek Microplate Reader (detail method is provided in supplementary information). Approximately, 20 and 100 *μ*g of protein, respectively, from the yeast and animal samples were separated by 12% or 13% SDS-PAGE and transferred to PVDF membranes. These membranes were then incubated with primary antibodies specific to GFP (1 : 1000) (^#^598, Medical & Biological Laboratories, Nagoya, Japan), *β*-actin (^#^CW0096, CoWin Biotech, Beijing, China), microtubule-associated protein 1 light chain 3B (LC3B) (1 : 1000) (^#^2775 s, Cell Signaling Technology, Boston, USA), or glyceraldehyde 3-phosphate dehydrogenase (^#^CW0100a, Beijing ComWin Biotechnology, Beijing, China) for 1 h, followed by secondary antibodies (1 : 5000) (horseradish peroxidase-linked anti-rabbit (^#^CW0103) or anti-mouse IgGs (^#^CW0102) (CoWin Biotech, Beijing, China). The antigens were visualized by using the ECL Western Blot Kit (CoWin Biotech, Beijing, China), whereas the protein bands were analyzed by using the ImageJ software (National Institute of Health, Rockville, MD, United States).

### 2.14. Statistical Analysis

The data were expressed as mean ± SEM values of three independent experiments and subjected to one-way ANOVA and Dunnett's multiple comparison tests or log-rank (Mantel-Cox) test by using the GraphPad Prism software. Statistical significance was established at *p* < 0.05.

## 3. Results

### 3.1. EHR Extension of the Lifespan

**Figure 1:**
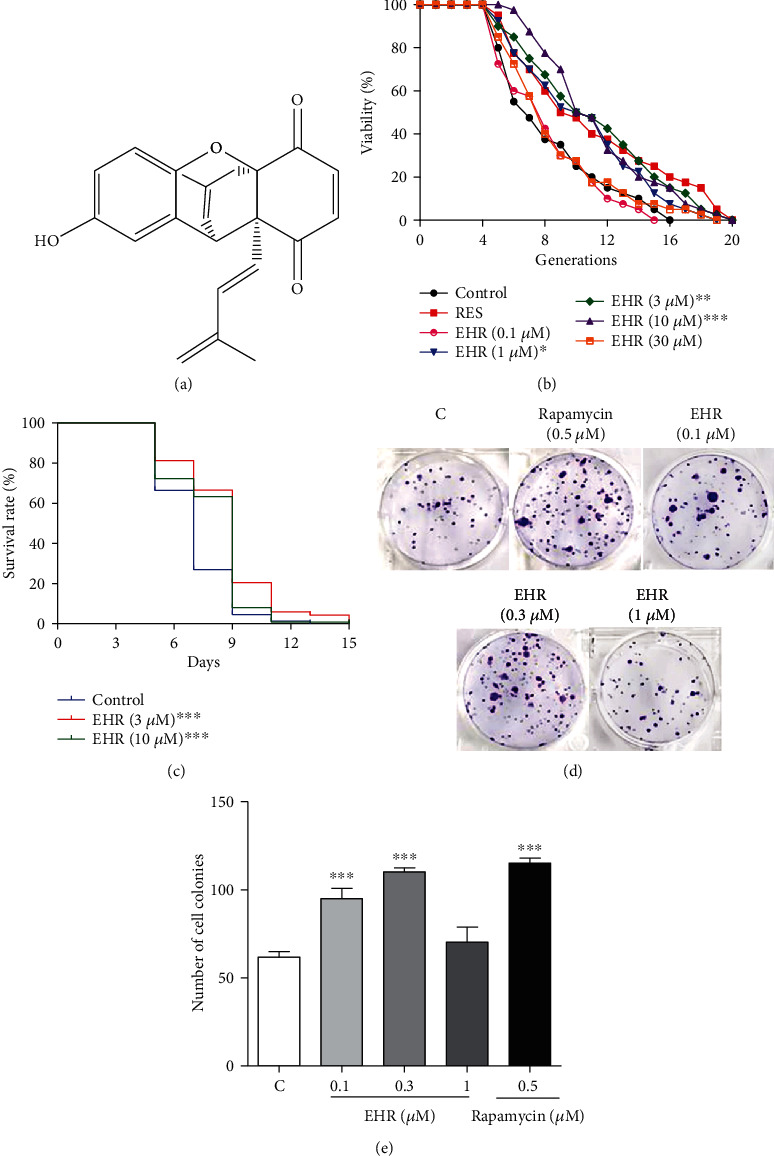
The chemical structure and effect of EHR on the lifespan of yeasts and mammals. (a) The chemical structure of EHR. (b) The replicative lifespan of K6001 yeast after treating EHR. The mean lifespan of each treatment group is as follows: control (7.42 ± 0.54), RES at 10 *μ*M (10.20 ± 0.77^∗∗^), EHR at 0.1 *μ*M (7.30 ± 0.48), EHR at 1 *μ*M (9.63 ± 0.64^∗^), EHR at 3 *μ*M (10.33 ± 0.70^∗∗^), EHR at 10 *μ*M (10.58 ± 0.57^∗∗∗^), and EHR at 30 *μ*M (7.78 ± 0.54). (c) The chronological lifespan of YOM36 after treating EHR. The mean lifespan of each treatment group is as follows: control (6.99 ± 0.15), EHR at 3 *μ*M (8.57 ± 0.22^∗∗∗^), and EHR at 10 *μ*M (7.95 ± 0.20^∗∗∗^). (d) The survival colony-forming units (CFUs) of PC12 cells. (e) Corresponding number of cell colonies in (d). Each assay was performed thrice, and the sample numbers of each group were six. In the present study, one-way ANOVA and Dunnett's multiple comparison tests were used for replicative lifespan and yeast-like chorological lifespan assay. The log-rank (Mantel-Cox) test was used for the yeast chorological lifespan assay. ^∗^, ^∗∗^, and ^∗∗∗^ represent significant differences from the control group at *p* < 0.05, *p* < 0.01, and *p* < 0.001, respectively. The replicative lifespan assay needed 4 days; the chronological lifespan assay needed 15-20 days as well as the yeast-like chronological lifespan assay needed 22 days.

A series of benzoquinone-type molecules with antiaging activity were isolated from *O. bracteatum* in a previous study based on the K6001 yeast bioassay system [23]. Among these molecules, EHR (Figure 1(a)) demonstrated the best performance in extending the replicative lifespan of K6001 at 1, 3, and 10 *μ*M (Figure 1(b)). Thus, this molecule is a valuable substance for intensive studies. The chronological lifespan of YOM36 after treatment with EHR was then studied. The survival time of EHR-treated groups was 15 days, which was significantly longer than that of the control group (13 days) at concentrations of 3 and 10 *μ*M (Figure 1(c)). Although yeast has many vital features that are evolutionally conserved in mammal cells, the antiaging activity of EHR in mammal cells must be confirmed to evaluate the antiaging activity of this molecule. Therefore, the conducted studies have examined the effect of EHR on the yeast-like chronological lifespan of PC12 cells and used rapamycin as a positive control. EHR can significantly increase the viability of PC12 cells at concentrations of 0.1 and 0.3 *μ*M compared with the control group (Figures 1(d) and 1(e), respectively). These results generally demonstrate the antiaging effects of EHR on yeast and mammals.

### 3.2. Antioxidative Stress Activity of EHR

**Figure 2:**
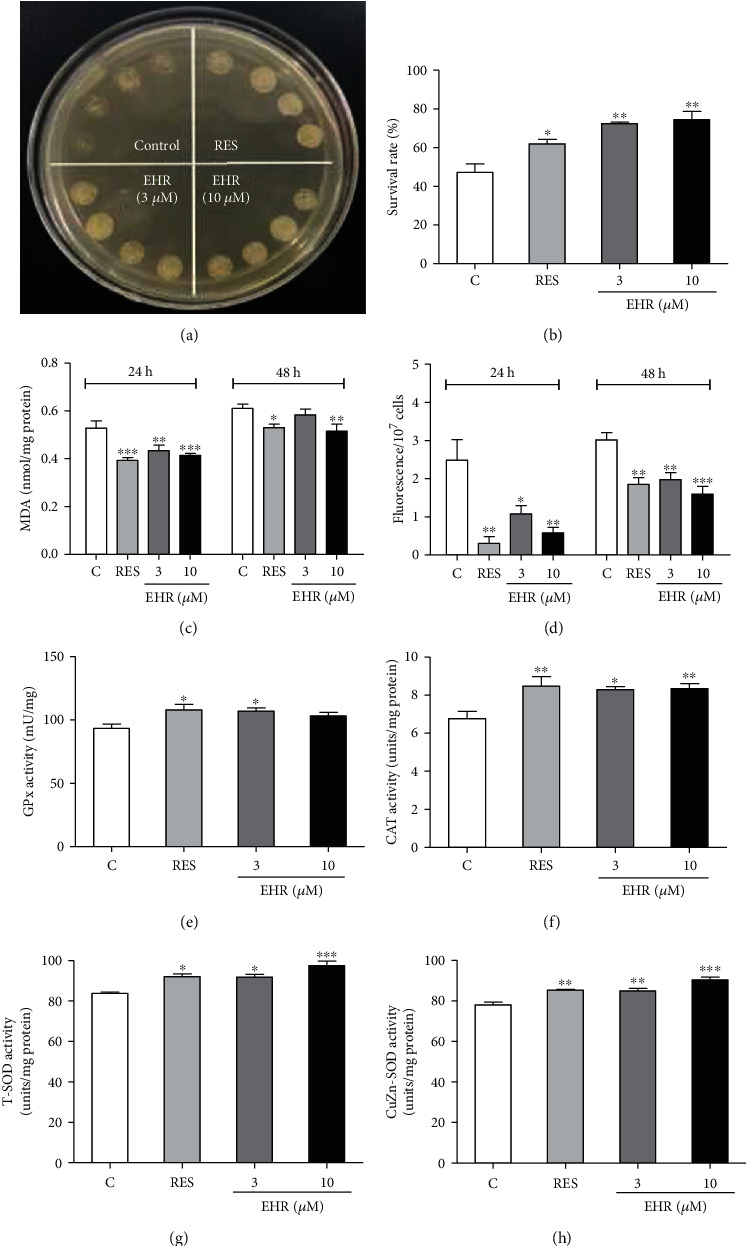
The antioxidant activity of EHR in yeast. (a) Qualitative survival condition of yeast under H_2_O_2_. (b) Quantitative survival rate of yeast under H_2_O_2_ (^∗^*p* < 0.05 and ^∗∗^*p* < 0.01). (c) The MDA level of yeast in 24 h and 48 h (^∗^*p* < 0.05, ^∗∗^*p* < 0.01, and ^∗∗∗^*p* < 0.001). (d) ROS level of yeast was represented by the fluorescence intensity of 1 × 10^7^ yeast cells in 24 h and 48 h (^∗^*p* < 0.05, ^∗∗^*p* < 0.01, and ^∗∗∗^*p* < 0.001). (e) GPx activity of yeast (^∗^*p* < 0.05). (f) CAT activity of yeast (^∗^*p* < 0.05 and ^∗∗^*p* < 0.01). (g, h) Total SOD activity and CuZn-SOD activity after treatment of EHR at 24 h (^∗^*p* < 0.05, ^∗∗^*p* < 0.01, ^∗∗∗^*p* < 0.001). Each assay was performed thrice, and the sample numbers of each group were five. One-way ANOVA and Dunnett's multiple comparison tests were used to analyze the results of each experiment.

Oxidative stress, which is a crucial factor in the advancement of the aging process, is caused by excessive levels of ROS; these levels cannot be resisted by the antioxidant system. This stress can damage DNAs, proteins, and lipids and impair physiological functions [28]. The survival capability of yeast under H_2_O_2_ was evaluated to highlight the important role of antioxidative stress in the antiaging effect of EHR. Differences were initially observed in the survivability of each group under oxidative stress (Figure 2(a)). Moreover, after a certain number of these cells were cultured with H_2_O_2_, the antioxidant activity of EHR was quantitatively identified and compared with RES as a positive control (Figure 2(b)) according to the survival rate of yeast cells. The survival capability of yeast significantly increased and became comparable with that of the positive control after EHR treatment.

In the mitochondrial electron transport chain, ROS is generated as a byproduct involved in the aging process [29]. The ROS at low concentrations can maintain a normal cellular metabolism but may fatally damage cells, tissues, and organisms at abnormally high concentrations [5]. MDA, as an index of membrane lipid peroxidation, is also produced by ROS during lipid reaction. Therefore, ROS and MDA levels in yeast have been studied to determine the degree of intracellular oxidation. At 24 h, EHR significantly reduced the MDA level in yeast at concentrations of 3 and 10 *μ*M; at 48 h, EHR significantly reduced the MDA level at a concentration of 10 *μ*M (Figure 2(c)).  Figure 2(d) shows that the fluorescence intensity of yeast significantly decreased after EHR treatment at 24 and 48 h. Therefore, EHR can constantly abate the ROS and MDA levels of yeast, thereby demonstrating its antioxidative stress activity.

Antioxidant enzymes, including CAT, GPx, and SOD, are important components in the antioxidant system of organisms. CAT catalyzes the decomposition of hydrogen peroxide, GPx breaks down the peroxide in organisms to prevent oxidative stress, and SOD catalyzes O_2_^−^ to less toxic hydrogen peroxide [28, 30]. Therefore, the enzymatic activities of CAT, GPx, and SOD were measured in this work. A significant increase in the total GPx and CAT activities was observed in EHR-treated groups as shown in Figures 2(e) and 2(f), while total SOD and CuZn-SOD activities were significantly improved after treatment of EHR at doses of 3 and 10 *μ*M for 24 h, respectively (Figures 2(g) and 2(h)). This finding suggests that EHR counters oxidative stress by increasing the activities of CAT, GPx, and SOD. Overall, EHR significantly reduces ROS and MDA levels while increasing CAT, total GPx, and SOD activities to protect cells from oxidative stress, thereby highlighting its antioxidative stress activity.

### 3.3. EHR cannot Extend the Replicative Lifespan of Oxidative Stress-Related Yeast Mutants

**Figure 3:**
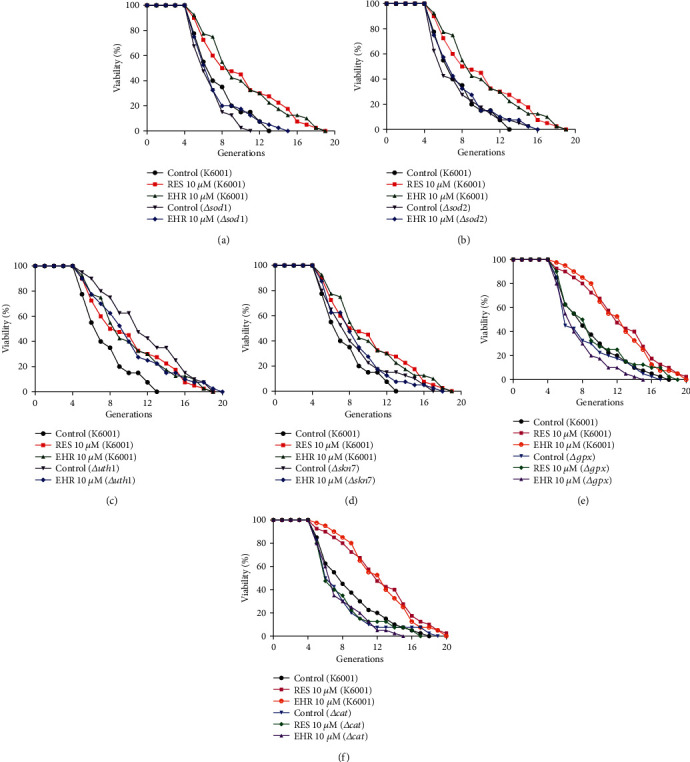
The effects of EHR on the replicative lifespan of *Δsod1* (a), *Δsod2* (b), *Δuth1* (c), *Δskn7* (d), *Δgpx* (e), and *Δcat* (f) with a K6001 background. The average lifespan of K6001 in the control group was 6.58 ± 0.38, whereas those of RES and EHR at 10 *μ*M were 9.1 ± 0.67 (*p* < 0.01) and 9.2 ± 0.64 (*p* < 0.001), respectively. (a) The average lifespan of *Δsod1* in the control group was 5.78 ± 0.28, whereas that of EHR at 10 *μ*M was 6.43 ± 0.43. (b) The average lifespan of *Δsod2* in the control group was 6.45 ± 0.48, whereas that of EHR at 10 *μ*M was 6.43 ± 0.45. (c) The average lifespan of *Δuth1* in the control group was 10.58 ± 0.66 (*p* < 0.001), whereas that of EHR at 10 *μ*M was 9.15 ± 0.63. (d) The average lifespan of *Δskn7* in the control group was 7.65 ± 0.57, whereas that of EHR at 10 *μ*M was 7.6 ± 0.51. In the lifespan assay of *Δgpx and Δcat,* the average lifespan of K6001 in the control group was 7.98 ± 0.58, whereas those of RES and EHR at 10 *μ*M were 11.50 ± 0.69 (*p* < 0.001) and 11.48 ± 0.59 (*p* < 0.001), respectively. (e) The average lifespan of *Δgpx* in the control group was 7.28 ± 0.56, whereas those of RES and EHR at 10 *μ*M were 8.10 ± 0.61 and 6.65 ± 0.41, respectively. (f) The average lifespan of *Δcat* in the control group was 6.73 ± 0.46, whereas those of RES and EHR at 10 *μ*M were 6.85 ± 0.50 and 6.73 ± 0.42. Each experiment was repeated thrice.

As an antioxidant enzyme, SOD can effectively remove superoxide anion radicals to counteract oxidative stress, and the overexpression of SOD encoded by *SOD1* and *SOD2* genes can extend cellular longevity [31, 32]. *UTH1* is a gene that can respond to oxidative stress, and its deletion extends the lifespan of yeast [33]. Skn7 is a major transcription factor that controls the oxidative stress response of *Saccharomyces cerevisiae* [34]. Replicative lifespan assays of these mutants were performed to understand the relationship between the antiaging effects of EHR and those of *SOD1*, *SOD2*, *GPx*, *CAT*, *UTH1*, and *SKN7* genes. Consistent with previous reports, Figure 3 shows that the replicative lifespan of *Δsod1*, *Δsod2*, *Δgpx*, and *Δcat* with a K6001 background is similar to that of wild-type K6001, whereas the replicative lifespan of the *Δuth1* with a K6001 background is longer than that of wild-type K6001 [33–35]. Furthermore, EHR cannot extend the replicative lifespan of *Δsod1*, *Δsod2*, *Δgpx*, *Δcat*, *Δuth1*, and *Δskn7* with a K6001 background (Figure 3). In other words, *SOD1*, *SOD2*, *GPx*, *CAT*, *UTH1*, and *SKN7* genes are involved in the antiaging effect of EHR.

### 3.4. EHR Induces Autophagy in Yeast

**Figure 4:**
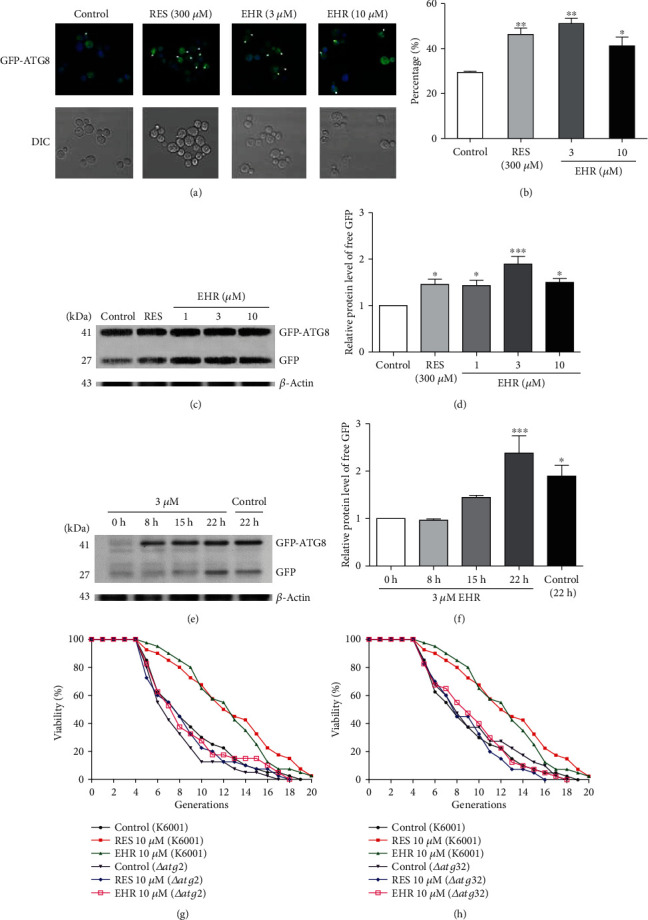
Effects of EHR on the autophagy of yeast. (a) The merge fluorescence images of DAPI and GFP and the image of YOM38-*GFP-atg8* yeast after EHR treatment. (b) The percentage of yeast cells containing autophagosome in the observation cells (^∗^*p* < 0.05 and ^∗∗^*p* < 0.01). (c, e) Western blot analysis results for the free GFP of yeast in dose and time courses. (d, f) The digital results of (c) and (e), respectively (^∗^*p* < 0.05 and ^∗∗∗^*p* < 0.001). (g, h) The changes on replicative lifespan of *Δatg2* and *Δatg32* of yeast. The average lifespan of K6001 in the control group was 8.05 ± 0.59, whereas those of RES and EHR at 10 *μ*M were 11.8 ± 0.73 (^∗∗∗^*p* < 0.001) and 11.6 ± 0.60 (^∗∗∗^*p* < 0.001), respectively. (g) The average lifespan of *Δatg2* in the control group was 6.78 ± 0.45, whereas those of RES and EHR at 10 *μ*M were 7.53 ± 0.56 and 7.78 ± 0.60, respectively. (h) The average lifespan of *Δatg32* in the control group was 8.40 ± 0.64, whereas those of RES and EHR at 10 *μ*M were 7.75 ± 0.50 and 8.48 ± 0.58, respectively. Each experiment was repeated thrice, and the sample numbers of each group were four. One-way ANOVA and Dunnett's multiple comparison tests were used to analyze the results of each experiment.

The autophagy level decreases with aging; specifically, inducing autophagy shows a potent antiaging effect, whereas decreasing autophagy can accelerate aging [36]. In yeast and mammalian cells, Atg8 and LC3B are molecular markers for monitoring the autophagic process. The Atg8 conjugated to phosphatidylethanolamine is localized to a preautophagosomal structure, which is crucial in autophagosome formation. Therefore, Atg8 plays an important role in measuring the autophagy level [37]. The YOM38 yeast strain, which expresses *GFP-Atg8*, was used in this study to monitor the autophagy level via fluorescence microscopy.  Figure 4(a) presents the fluorescent images of GFP in autophagosome in yeast, while Figure 4(b) presents the statistical results. EHR significantly increased the percentage of cells with green fluorescence at concentrations of 3 and 10 *μ*M. The EHR concentration at 3 *μ*M showed a better effect than that of 10 *μ*M EHR. The autophagy flux represents the entire autophagy process, and free GFP was eventually released in the vacuole. Therefore, the expression of free GFP was analyzed using western blot to further confirm the autophagy level. As shown in Figures 4(c) and 4(d) and S1a, EHR significantly enhanced the level of free GFP at 1, 3, and 10 *μ*M, respectively, among which the EHR at 3 *μ*M exhibited the best effect. Therefore, the EHR at a concentration of 3 *μ*M was used to examine the time-dependent behavior of GFP after treatment. The released GFP is time-dependent within the tested time range (Figures 4(e), 4(f), and S1b), and *ATG2* and *ATG32* are two of the most important genes that mediate autophagy in yeast. Therefore, the replicative lifespan assay of *Δatg2* and *Δatg32* of yeast with a K6001 background was further performed. EHR did not affect the replicative lifespan of these mutants (Figures 4(g) and 4(h)), thereby confirming the involvement of autophagy in the antiaging activity of EHR in yeast.

### 3.5. EHR Induces Autophagy in Mice

**Figure 5:**
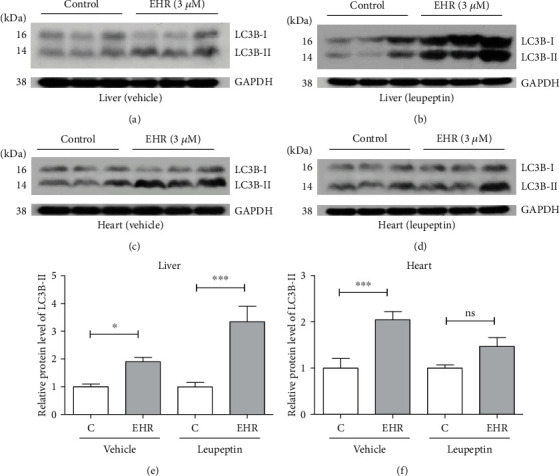
The effects of EHR on the autophagy in mice in vivo. (a, b) The LC3B protein level in the mice liver after administrating EHR and EHR plus leupeptin, respectively. (c, d) The LC3B protein level in the mice heart after administrating EHR and EHR plus leupeptin, respectively. (e) The digital results of western blotting analysis in (a) and (b). (f) The digital results of western blotting analysis in (c) and (d). Each experiment was repeated thrice, and the sample numbers of each group were three. ^∗^ and ^∗∗∗^ represent significant differences from the control group at *p* < 0.05 and *p* < 0.001, respectively. One-way ANOVA and Dunnett's multiple comparison tests were used to analyze the results of each experiment.

Autophagy is also important in determining the lifespan of some organisms [38]. Compared with yeast, which is a unicellular eukaryote, mice have 30,000 protein-coding genes similar to humans, and approximately 80% of the human genome has a single identifiable ortholog in mouse genes [39]. Hence, using mice can provide highly reliable evidence. Microtubule-associated protein 1 LC3B in mammal cells is a homologous protein of Atg8 to form autophagosome [40]. This protein started to appear in the cytoplasm as LC3B-I and transformed into LC3B-II during autophagy. Leupeptin, one kind of autophagy inhibitor, can impede autophagosome degradation. This inhibitor is an effective tool for blocking the autophagy flux when determining the actual autophagy level. Therefore, leupeptin was used to further investigate the change in the autophagy level after EHR treatment. In the experiment, intraperitoneal injection of EHR was administered among mice at 3 *μ*M for 6 h, and the mice were treated with or without leupeptin after injection of EHR for 4 h. EHR significantly promoted the expression of LC3B-II in the liver and heart of mice without leupeptin (Figures 5(a), (c), (e), and (f) and S2). Interestingly, the expression of LC3B-II in the liver after treatment with leupeptin significantly increased with EHR treatment (Figures 5(b), (e), and S2). However, such an expression did not change in the heart (Figures 5(d), (f), and S2). Therefore, EHR significantly induces autophagy in the liver of mice.

### 3.6. Effect of EHR on the SIR2 Gene Expression and the Chronological Lifespan of *Δ*sir2 and *Δ*uth1 of Yeast with YOM36 Background

**Figure 6:**
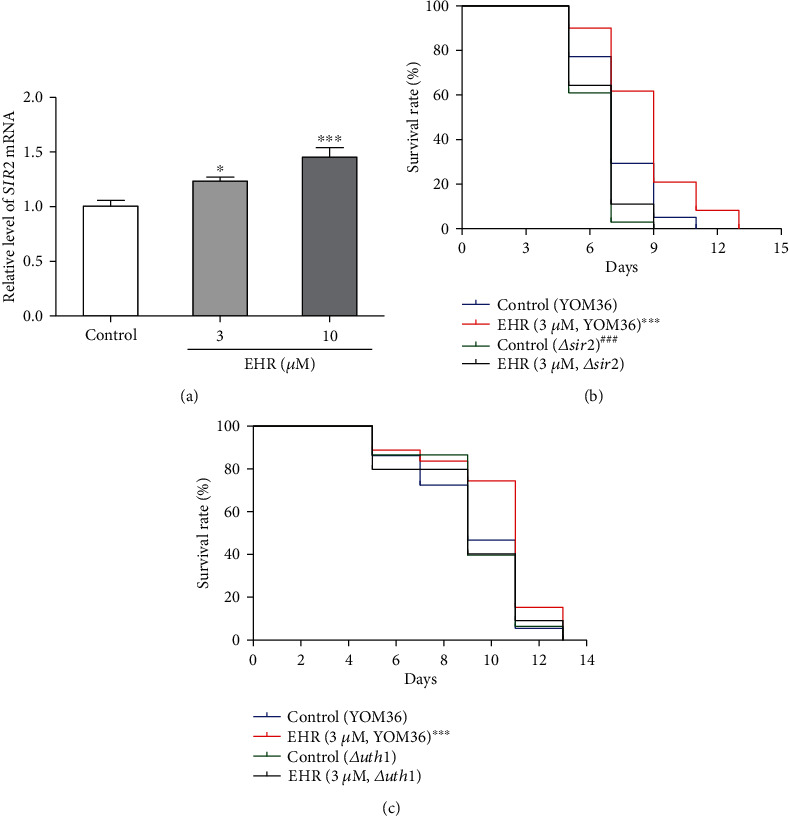
The effects of EHR on the *SIR2* gene expression of yeast (a), the chronological lifespan of *Δsir2* (b) and *Δuth1* (c) of yeast with the YOM36 background. This assay was taken thrice, and the sample numbers of each group were five or three. The log-rank (Mantel-Cox) test was used for the yeast chorological lifespan assay. ^∗^ and ^∗∗∗^ indicate a significant difference between the control group and EHR-treated group at *p* < 0.05 and *p* < 0.001, whereas ^###^ indicates a significant difference between the *Δsir2* control group and YOM36 control group at *p* < 0.001.

The increase in *SIR2* and the reduction of the *UTH1* gene expression can extend replicative lifespan because Sir2 is considered to be a prolongevity factor, while the deficiency of the *UTH1* gene can prolong the replicative lifespan in yeast [10, 33]. However, the similarities in the roles of these genes in the chronological lifespan in yeast remain uncertain. The *SIR2* gene expression analysis and chronological lifespan assay of *Δ*s*ir2* and *Δuth1* with a YOM36 background were performed to understand the effect of EHR on the *SIR2* gene and the relationship between *SIR2* and *UTH1* genes. A significant increase in the *SIR2* gene expression in the EHR treatment group was observed compared with the control group at doses of 3 and 10 *μ*M (Figure 6(a)). The chronological lifespan of *Δsir2* with a YOM36 background was significantly shorter than that of the YOM36 yeast (Figure 6(b)). This finding highlights the importance of the *SIR2* gene in extending the chronological lifespan of yeast. The EHR treatment at 3 *μ*M significantly extended the chronological lifespan of YOM36 from 11 days to 13 days. However, EHR did not increase the chronological lifespan of *Δsir2 and Δuth1* with the YOM36 background (Figures 6(b) and 6(c), respectively). However, the extension of the chronological lifespan of *Δuth1* with the YOM36 background was not observed in Figure 6(c). This finding suggests the involvement of *SIR2* and *UTH1* genes in the antiaging effect of EHR for the chronological lifespan of yeast. *UTH1* genes did not take important roles in the chronological lifespan of YOM36 yeast but were required to participate in the antiaging effect of EHR.

## 4. Discussion

The previous study conducted by the authors found a series of antiaging benzoquinone-type molecules from *O. Bracteatum* and determined the chemical structure of these compounds [23]. Among these molecules, EHR not only has significant antiaging activities but also emerges as the best molecule. Therefore, further studies must focus on the antiaging effects and mechanism of the EHR action. The lifespan assay results (Figures 1(b) and 1(c)) of yeast and the yeast-like chronological lifespan in PC12 cells (Figures 1(d) and 1(e)), respectively, suggest that EHR has an antiaging effect for yeast and mammal cells.

Oxidative stress plays a crucial role in the aging process [5]. Previous studies revealed the isolation of many natural products with antioxidative stress properties from food and herb medicines [16, 17, 35]. Therefore, the current study focused on the antioxidative stress of EHR. The significant increase in survival rates under oxidative conditions (Figures 2(a) and 2(b)), the GPx, CAT, and SOD enzyme activities (Figures 2(e), 2(f), 2(g), and 2(h), respectively) and the reduction in MDA and ROS levels (Figures 2(c) and 2(d), respectively) indicate the involvement of antioxidative stress in the antiaging effect of EHR. The lifespan assays of *Δsod1*, *Δsod2*, *Δgpx*, *Δcat*, *Δuth1*, and *Δskn7* with a K6001 background, which all had related effects on antioxidative stress, were performed to obtain additional evidence to support the present hypothesis. The changes in the lifespan of these mutants in Figure 3 suggest that *SOD1*, *SOD2*, *GPx*, *CAT*, *UTH1*, and *SKN7* contribute to the antiaging effect of EHR. Interestingly, all the natural products from previous studies have the function of antioxidative stress despite differences in structural features [16–20, 35]. Therefore, the antioxidant properties of these molecules will be examined in the future.

Autophagy takes an important role in removing damaged molecules, and enhanced autophagy can extend the lifespan of yeasts, worms, flies, and mice [38]. A previous study indicated that autophagy was involved in the antiaging activity of cucurbitacin B [16]. Therefore, the effects of EHR on autophagy in yeasts and mammals were also investigated. The increase in free GFP in YOM38-*GFP-ATG8* yeast (Figures 4(a)–(f)), the absence of changes in the replicative lifespan of *Δatg2* and *Δagt32* of yeast (Figures 4(g)–(h)), and the increase in LC3B-II of the liver and heart in mice (Figure 5) after treatment of EHR confirmed that autophagy is crucial in the antiaging activity of EHR. However, opposite findings were obtained for the autophagy of the liver and heart of mice. Specifically, the EHR-induced LC3B-II protein level in the heart was higher than that in the liver. The significant changes in the EHR-induced LC3B-II in the heart were diminished after the leupeptin administration, but the opposite was observed for the expression of LC3B-II in the liver.

*SIR2* is a longevity gene in which deletion or overexpression can shorten or prolong the replicative lifespan of yeast [10]. A previous study found that *SIR2* and *UTH1* genes interacted with each other, and deletion of the latter extends the replicative lifespan of yeast [17]. The *SIR2* gene expression was investigated to determine the involvement of these genes in the chronological lifespan of yeast and their contributions to the antiaging effect of EHR. *Δsir2* and *Δuth1* with a YOM36 background were also constructed for a chronological lifespan assay. The changes in the *SIR2* gene expression (Figure 6(a)) and the chronological lifespan of *Δsir2* with the YOM36 background (Figure 6(b)) suggest the involvement of the *SIR2* gene in the chronological lifespan of yeast and the antiaging effect of EHR. However, some reports showed that *Δsir2* can extend the chronological lifespan [11], but other studies did not find any differences [12]. Such discrepancies can be ascribed to the usage of different yeast strains and media in these studies. Meanwhile, the deletion of the *UTH1* gene can extend the replicative lifespan of K6001 but does not affect the chronological lifespan of YOM36 (Figures 3(c) and 6(c)). This finding suggests that the *UTH1* gene can be ignored in the chronological lifespan of YOM36. However, the *UTH1* gene is required to participate in the antiaging effect of EHR.

## 5. Conclusion

Overall, EHR produced antiaging effects on yeast and mammal cells by inducing autophagy and antioxidative stress. Several well-known antiaging molecules, such as RES, nicotinamide mononucleotide, and rapamycin, have been recently reported to ease the symptoms of type 2 diabetes, Alzheimer's disease, cancer, cardiovascular diseases, and obesity [41]. Therefore, antiaging substances may have potential applications in the treatment of age-related illnesses. The new function and mechanism of EHR in various age-related pathological models and the safety evaluation of EHR must be examined in the future to develop EHR as a drug or health food.

## Data Availability

All the figures and table used to support the findings of this study are included within the article.

## Supplementary Materials

Supplementary Materials Figure S1: origin data of western blotting analysis of free GFP in yeast in Figures 4(c) and 4(e). Figure S2: origin data of western blotting analysis of LC3B-II in the liver and heart of mice in Figures 5(a), 5(b), 5(c) and 5(d).
