# Supplementary material for: Ehretiquinone from Onosma bracteatum Wall Exhibits Antiaging Effect on Yeasts and Mammals through Antioxidative Stress and Autophagy Induction
Source: Oxid Med Cell Longev. 2021 Jan 13;2021:5469849. doi: 10.1155/2021/5469849 (PMC7822689; doi:10.1155/2021/5469849)
Supplement: Supplementary Materials — Figure S1: origin data of western blotting analysis of free GFP in yeast in Figures 4(c) and 4(e). Figure S2: origin data of western blotting analysis of LC3B-II in the liver and heart of mice in Figures 5(a), 5(b), 5(c) and 5(d). [file 5469849.f1.docx]

**Supplementary information**

**Materials and Methods**

*Determination of Protein Concentration.*

At first, the yeast cells after sonication were centrifuged at 12000 g / min for 10 min. The supernatant as protein samples were taken and diluted with PBS for 10 times to determine the protein concentration, respectively. Afterwards, the bovine serum albumin (BSA) as standard sample in BCA protein assay kit (Cowin Biotechnology, Beijing) was diluted different concentrations at 0, 0.0625, 0.125, 0.25, 0.5, 1 and 2 mg/ml. Meanwhile, BCA working solution was prepared with the ratio of working solution A: B = 50:1. The 200 μl BCA working solution and 25 μl each standard or each sample were added into each well of 96 wells plate, respectively. Each sample was repeated for two times and mixed well. Subsequently, the 96-wells plate was incubated 37^o^C for 25 minutes and measured the absorbance of BSA standard and protein samples at 562 nm of wavelength with the BioTek Microplate Reader. After that, the standard curve and calculation formula of protein concentration were gotten. The sample protein concentrations were calculated with this formula, respectively.

*Measurement of, GPx, CAT and SOD Enzymatic Activity in Yeast.*

For GPx enzyme activity assay, the 2 μg protein of each sample was taken and the total glutathione enzyme assay kit was used to determine glutathione enzyme activity. The general protocol is as follows: the glutathione peroxidase detection solution, samples, GPx detection working solution and peroxide reagent were added in 96 well plate in proper order. The absorbance value at A340 value every 5 minutes was measured for six times after mixing. The total glutathione activity was calculated according to the formula. The activity of GPx in the detection system was = [Δ A340 (sample) - Δ A340 (blank)] / (0.00622 × 0.276). Total glutathione peroxidase activity in the sample = total glutathione peroxidase activity in the detection system × dilution ratio / sample protein concentration.

For CAT enzyme activity assay, the 5 μg protein of each sample was taken and the catalase assay kit was used to measure catalase activity. The general operation is as follows: first, took different concentrations of hydrogen peroxide solution, added color working solution and reacted at 25 ^o^C for 15 minutes, then measured the absorption value of A250, and determined the standard curve of hydrogen peroxide concentration. Afterwards, added catalase buffer and 250 mM hydrogen peroxide in each sample. After reaction at 25 ^o^C for 1-5 minutes, added the enzyme reaction termination solution to terminate the reaction, then added the color working solution, reacted at 25 ^o^C for 15 minutes and measured the absorption value of A520. The concentration of hydrogen peroxide in the reaction system was calculated, and calculate the catalase activity was calculated as following. Sample catalase activity = [consumption of micromole of hydrogen peroxide] × [dilution ratio] / ([reaction minutes] × [sample volume] × [protein concentration]), [consumed micromole of hydrogen peroxide] = [blank control micromole of residual hydrogen peroxide] - [sample micromole of residual hydrogen peroxide]).

For SOD enzyme activity assay, the 2.5 μg protein of each sample was taken, and SOD enzyme assay kit was used to determine the activity of superoxide enzyme. The process is as follows: At first, each sample was mixed with reagent VII, reacted for 1min to lost the Mn-SOD enzyme activity in the samples and got the supernatant after centrifugation as samples. The reagent I, blank control, samples, and the samples treated by reagent VII were added to the 96 well plate, respectively. Afterwards, the reagent II, reagent III and reagent IV were added, mixed well and incubated at 37 ^o^C for 40 min. Final, the absorbance value of A550 of samples were measured after adding color reagent and standing at room temperature for 10 min. The activity of SOD enzyme was calculated according to the formula as following: [Control group OD value - Determination group OD value] / Control group OD value / 50% × total volume of reaction solution / sample volume / protein concentration of sample to be measured.

**Supplementary Figures:**

**
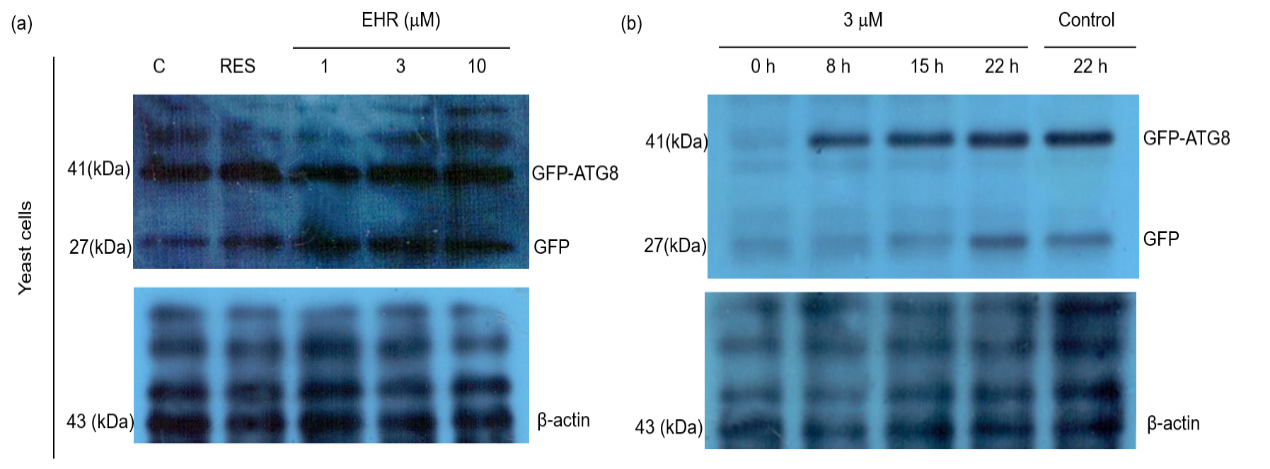
**

**Supplementary Figure S1:** Origin data of western blotting analysis of free GFP in yeast in Figure. 4c and 4e.

**
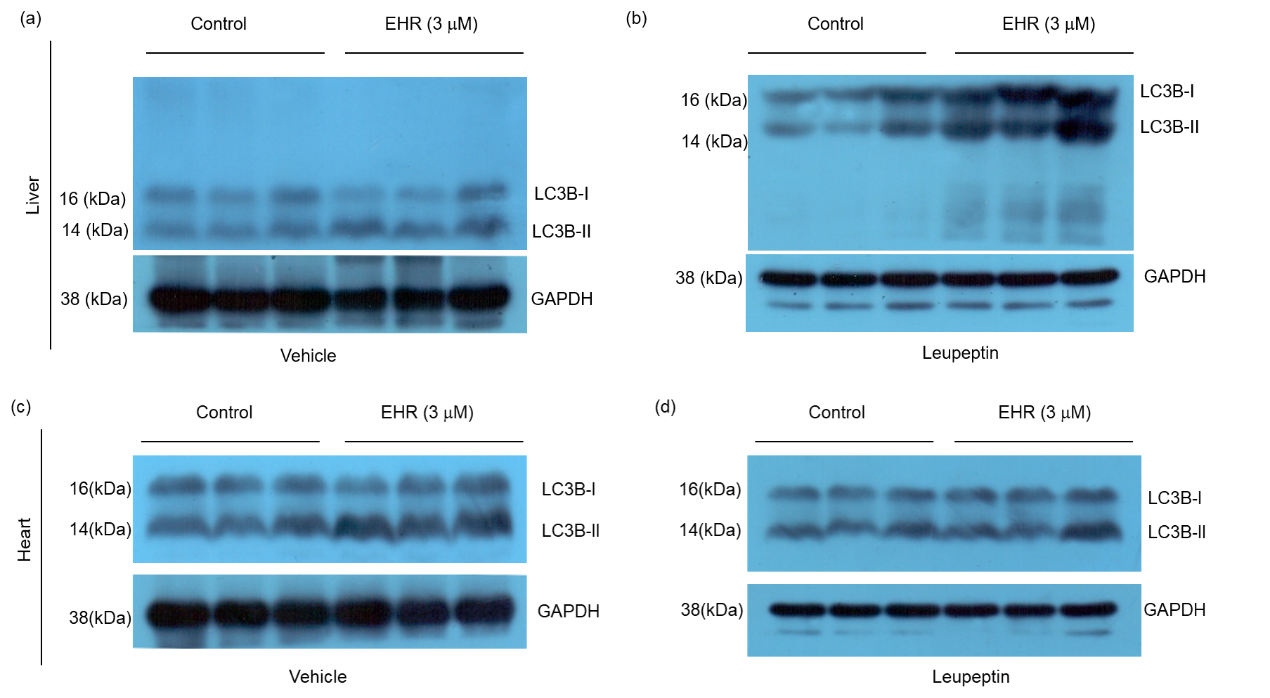
**

**Supplementary Figure S2:** Origin data of western blotting analysis of LC3B-II in liver and heart of mice in Figure. 5a, b, c and d.
